# The role of probiotics and prebiotics in modulating of the gut-brain axis

**DOI:** 10.3389/fnut.2023.1173660

**Published:** 2023-07-26

**Authors:** Fereshteh Ansari, Mehrdad Neshat, Hadi Pourjafar, Seid Mahdi Jafari, Shohreh Alian Samakkhah, Esmaeel Mirzakhani

**Affiliations:** ^1^Razi Vaccine and Serum Research Institute, Agricultural Research, Education and Extension Organization (AREEO), Tehran, Iran; ^2^Research Center for Evidence-Based Medicine, Health Management and Safety Promotion Research Institute, Tabriz University of Medical Sciences, Tabriz, Iran; ^3^Iranian EBM Centre: A Joanna Briggs Institute Affiliated Group, Tabriz, Iran; ^4^Department of Clinical Science, Faculty of Veterinary Medicine, Tabriz Medical Sciences, Islamic Azad University, Tabriz, Iran; ^5^Alborz University of Medical Sciences, Dietary Supplements and Probiotic Research Center, Karaj, Iran; ^6^Department of Food Materials and Process Design Engineering, Gorgan University of Agricultural Sciences and Natural Resources, Gorgan, Iran; ^7^Department of Analytical Chemistry and Food Science, Faculty of Science, Universidade de Vigo, Nutrition and Bromatology Group, Ourense, Spain; ^8^College of Food Science and Technology, Hebei Agricultural University, Baoding, China; ^9^Department of Food Hygiene and Quality Control, Faculty of Veterinary Medicine, Amol University of Special Modern Technologies, Amol, Iran; ^10^Department of Food Science and Technology, Faculty of Nutrition and Food Sciences, Tabriz University of Medical Sciences, Tabriz, Iran

**Keywords:** probiotics, prebiotics, gut microbiome, gut-brain axis, mental disorders, Alzheimer’s, depression, anxiety

## Abstract

Pro-and prebiotics have been indicated to modulate the gut-brain axis, which have supportive impacts on central nervous systems, and decrease or control the incidence of some mental disorders such as depression, anxiety, autism, Schizophrenia, and Alzheimer’s. In this review, complex communications among microbiota, gut, and the brain, and also recent scientific findings of the impacts and possible action mechanisms of pro-and prebiotics on mental disorders have been discussed. The results have shown that pro-and prebiotics can improve the function of central nervous system and play an important role in the prevention and treatment of some brain disorders; however, in order to prove these effects conclusively and firmly and to use these compounds in a therapeutic and supportive way, more studies are needed, especially human studies/clinical trials.

## 1. Introduction

The two-way communication between the gastrointestinal tract (GIT) and the brain has long been well known, with direct neural signals and indirect hormonal and enzymatic signals from the brain always being sent to the GIT lumen to control and regulate movement, secretion, and sensory transmission; on the other hand, similar signals are sent from the GIT to the brain affecting its functions and control and regulatory role of the brain. In fact, GIT is connected to the brain by about 200–600 million neurons (1, 2). In recent years, extensive studies have been conducted on the role and possible effects of the intestinal microbiome on brain functions as well as some central nervous system (CNS) disorders (3, 4). Mental diseases affect more than 1 billion people all over the world, and communal mental diseases refer to a range of depressive and anxiety disorders. According to the FAO/WHO, about 4.4 and 3.6% of the world’s population suffer from depressive and anxiety disorders, respectively (2).

Today, the use of natural supplements that strengthen the intestinal microbiome and ultimately have a positive effect on brain functions has received more attention from researchers. The use of pro-and prebiotic dietary supplements is one of the most popular products that have a positive effect on the intestinal microbiome, improving intestinal and gut-brain axis functions, with the potential and ability to play an effective role in preventing and treating some mental disorders (5). By definition, “probiotics” are living microorganisms that in sufficient quantities cause one or more beneficial effects on the host. The most important probiotics belong to the genera *Lactobacillus* and *Bifidobacterium.* Foods containing probiotics should comprise at least 7 log CFU cells and should be eaten at a rate of 100 g or mL per day to have effective influences on health and control and treatment of diseases (6–8).

Prebiotics are compounds indigestible by the human GIT (resistant to secretions and intestinal enzymes) that travel through the intestine and reach the colon intact. Prebiotics in the colon are broken down by the gut microbiome (GM) or probiotic microorganisms that are eaten together to produce beneficial compounds. In fact, the breakdown of prebiotics not only produces therapeutic and health-promoting compounds, but also strengthens and functions colon-based probiotics as a food source. The most important prebiotics belong to carbohydrates and the family of galactooligosaccharides (GOS), fructooligosaccharides (FOS), and xylooligosaccharides (2). National Health and Nutrition Examination Survey (NHANES) reported that people at least 20 years of age in the United States consume only 61% of their recommended level, while there is no official information on the consumption of prebiotics, there are recommendations from researchers like consuming 10 g of FOS or 7 g of GOS per day (9, 10). Prebiotics exert their effect in low doses, for example, the effective amount of polydextrose is about 2 to 7.5 g per day (11), resistant starch is 2.5 to 5 g per day (12), and inulin is 1 to 6 g per day (13).

The combined use of pro-and prebiotics, called synbiotics, has a synergistic effect and plays an important role in controlling and reducing the risk of some diseases, including mental disorders. In the absence of prebiotics, which are considered a food source for probiotics, the number of probiotics decreases, causing problems with the intestinal and general immune systems of the host, as well as causing some abnormalities such as constipation. On the other hand, if there are no probiotics or their number is significantly low, then prebiotics will play a lesser role in host health and disease control (14). Numerous studies have shown that pro-and prebiotics, together or alone, play an important role in neuroimmune processes. It has also been shown that their health effects on the CNS are related to the interactions between GM and colon-based probiotics, the immune and nervous systems, which occur through the secretion of certain enzymes, hormones, immunological factors, and neurotransmitters (5, 15, 16). Also, animal, clinical and paraclinical studies have shown that there is a relationship between the presence and activity of pro-and prebiotics in the gut, CNS and immune systems and eventually the incidence of Alzheimer’s, depression, schizophrenia, anxiety, autism, insomnia, severe stress, and other mental diseases (5, 17).

In this review, the possible role of pro-and prebiotics in regulating the immune and nervous systems, and finally the possible control and treatment of some mental disorders are discussed. The possible mechanisms involved in the healing process of CNS diseases by these supplements have also been investigated. Finally, the last part of this article provides an overview of the future prospects of using these compounds to treat mental disorders.

## 2. Gut microbiota and brain communications

It is recognized that the communication between the gut, the microbiota, and the brain is mediated by multiple signals from neural, immune, and endocrine pathways. The gut alone has a unique nervous system called the enteric nervous system (ENS), which is directly and permanently connected to the brain by the nerves. It is noteworthy that ENS is separated from the intestinal microbiota by the mucous cell layer; intestinal microbes do not have direct access to this local nervous system. It is possible that microbiota communicates indirectly with this nervous system by transmitting them from the intestinal lumen to the lamina propria via the microfold cells or dendritic cells, given the direct entry of resident microbes invasively causing ulceration and perforation in the intestine. Another possible communication pathway is intestinal bacterial secretions and metabolites such as short-chain fatty acids (SCFAs), exopolysaccharides (EPS), lipopolysaccharides (LPS), and glutamate that are able to cross the intestinal cell wall and directly affect the ENS, and are able to interact with some certain receptors; e.g., G-protein coupled receptors (GPCRs), and Toll-like receptors (TLRs) (3, 5).

**Figure 1:**
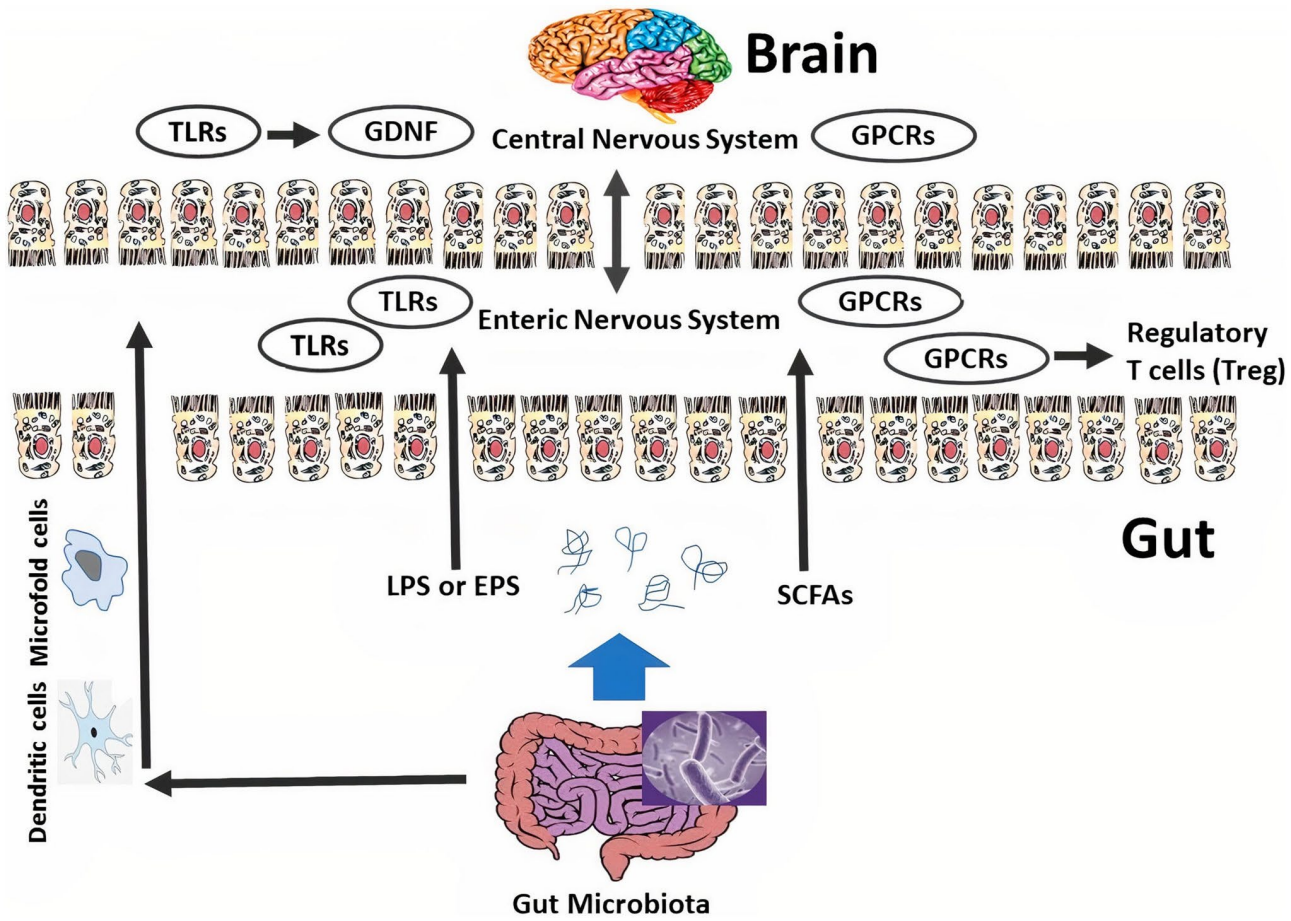
The connection among the microbiome, enteric nervous system/ENS, and central nervous system/CNS. Intestinal bacteria are transmitted from the intestinal lumen to the lamina propria by microfold cells and dendritic cells. Intestinal microbiome secretions like exopolysaccharides/EPS and short-chain fatty acids/SCFAs pass through the intestinal epithelium and directly affect the CNS. Intestinal bacteria and their metabolites can interact with certain receptors such as G-protein coupled receptors/GPCRs and Toll-like receptors/TLRs. The intestinal microbiome can adjust ENS function through TLRs, inspire the expression of glial cell line-derived neurotrophic factor/GDNF, and enhance the number of glial cells and enteric neurons. On the other hand, SCFAs adjust colonic regulatory T cell/cTreg homeostasis via affecting on GPCRs.

GPCRs are the receptors in the CNS, especially in the striatum, which play an important role in regulating and controlling metabolism and the inflammatory process in mental disorders. SCFAs produced by the activity of GM, especially in the presence of prebiotics, stimulate and activate GPCRs at the ENS and CNS (GPR109A, GPR41, and GPR43 recognized as SCFAs receptors), as depicted in Figure 1. For example, it has been shown that the secretion of SCFAs such as acetic, butyric, and propionic acids with an effect on GPR43 plays an important role in regulating T cell homeostasis and preventing colitis. Similar effects have been reported in the prevention of some mental disorders caused by damage and inflammation in the brain (3), which is described in Section 4 in detail.

TLRs are stimulated and activated by some secretions and metabolites of GM such as exopolysaccharides and LPS, and then the immune system and ENS release cytokines and neurohormonal mediators that strengthen the intestinal and nervous systems to prevent some intestinal and mental disorders (Figure 1). For instance, it has been indicated that the activity and secretion of microbiota with effect on TLR2 strengthen and regulate ENS integrity, stimulate the emergence of a glial cell line-derived neurotrophic factor, enhance the number of glial cells and enteric neurons, and ultimately survive and strengthen several kinds of neurons (3, 18).

## 3. Impacts of pro-and prebiotics on the CNS

**Figure 2:**
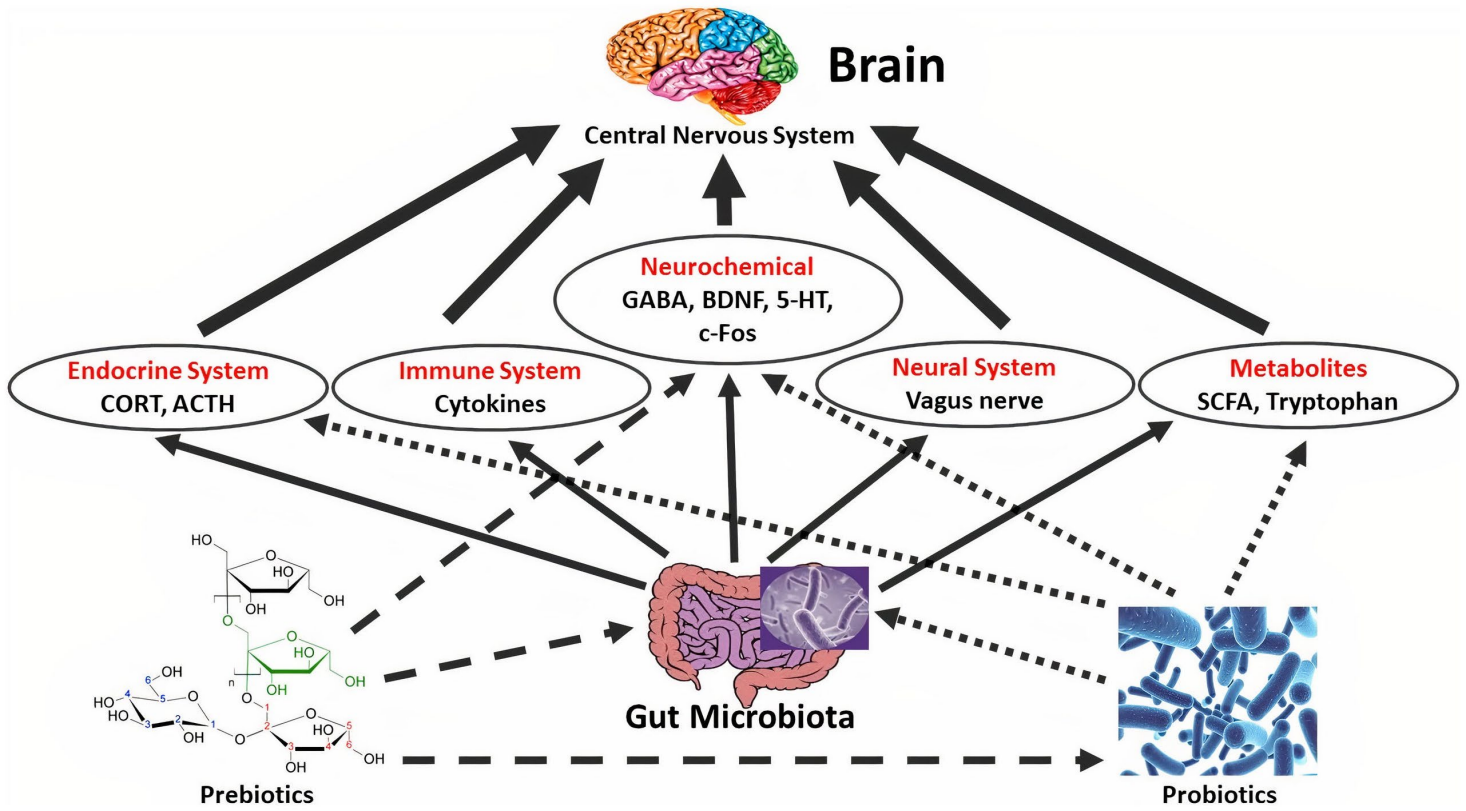
The effect of probiotics on the central nervous system/CNS through the effect on the microbiome-gut-brain axis. Probiotics affect brain function both directly and indirectly. Probiotic microorganisms affect the hypothalamic–pituitary–adrenal/HPA axis, through fluctuating corticosteroid/CORT and adrenocorticotropic hormone/ACTH ranges. The immune system is impacted by limited pro-inflammatory cytokine creation and inflammation and has stimuluses on the CNS. Probiotics can moreover straightly modify CNS biochemistry, for example by fluctuating 5-hydroxytryptamine/5-HT, brain-derived neurotrophic factor/BDNF, g-aminobutyric acid/GABA, Dopamine/DA, and c-Fos ranges, subsequently compelling mind and manners. The vagus and enteric nerves are also involved in gut-brain communications and are impacted by certain probiotic strains. Also, probiotic microorganisms regulate the gut microbiota by expanding microbiota variety and beneficial bacteria configuration. At that point, gut microbiota can adjust metabolites, such as, short-chain fatty acids/SCFAs, exopolysaccharides/EPS, and tryptophan, afterward, progresses CNS function indirectly. Also, the gut microbiota collaborates with the immune system, endocrine, and neural system.

Many studies have shown that GM can affect the gut-brain axis and play an important role in preventing and controlling some brain diseases such as Alzheimer’s, depression, and insomnia (4, 17–19). Although chemical drugs are used to treat these abnormalities, interest in applied studies and the use of natural compounds such as pro-and prebiotics, which have no specific side effects and have a preventive role, is increasing (Table 1). In fact, the presence of probiotics directly and by modulating the balance of intestinal microbiota toward positive function strengthens the gut-brain axis and has a positive effect on the treatment of some brain diseases. Also, the presence of prebiotics directly, and also, by modulating the balance of intestinal microbiota and strengthening and increasing the number of probiotics in the colon has the same therapeutic effect on mental disorders (2, 3, 5) (See Figure 2).

**Table 1:** Summary of recent systematic reviews on the effects of pre and probiotics on mental disorders.

Ref	Title	Covered articles	Search databases	Intervention and comparison	Quality assessment	Population	Outcome	Studies	Patients	Subclass of Outcome	Hetero-geneity (*L*^2^)	Data (95% confidence levels and *p* value)
(20)	From probiotics to psychobiotics – the gut-brain axis in psychiatric disorders	23	PubMed, Embase, Cochrane Central Register of Controlled Trials, and ClinicalTrials.gov	Pre or probiotic compared with placebo	Jadad scale	Diverse populations from students to the elderly and pregnant women	-A significant decrease in Beck depression inventory (BDI) scores after two months -Reduced depressive symptoms in patients with depression -Improvement of Hamilton Depression Rating Scale (HAM-D) scores	16	2,726	-	78%	−0.87 (−1.66–0.099)
(21)	Effect of Probiotics on Psychiatric Symptoms and CNS Functions in Human Health and Disease: A Systematic Review and Meta-Analysis	54	PubMed, Web of Science and Cochrane Library	Trials assessing the effectivity of viable and non-viable microorganisms or probiotics cell extracts with a blinded placebo control group	-	Adult men and women with different health conditions, including healthy people, people with mental disorders, or a specific functional CNS	- Reduction of depressive symptoms in both healthy and disordered groups	30	3,017	-	48%	−0.37 (−0.55, −0.20)
(22)	Effects of probiotics and paraprobiotics on subjective and objective sleep metrics: a systematic review and meta-analysis	15	PubMed (MEDLINE), Web of Science (via Thomas Reuters), Scopus and PsycINFO	Probiotics/ Paraprobiotics with placebo	Rosendale Scale, which combines the PEDro scale, Jadad scoring system, and Delphi List	People over 18 years who consumed probiotics	- Improving the quality of people’s sleep - A decrease in the Pittsburgh Sleep Quality Index (PSQI) score	11	452	-	58%	0.78 (0.395–1.166)
(23)	Efficacy of probiotics on stress in healthy volunteers: A systematic review and meta-analysis based on randomized controlled trials	25	Cochrane Library, Embase, Medline (Ovid), PsycINFO (Ovid), and CINAHL (EBSCOhost)	Probiotic compared with placebo	-	Participants with health conditions and no major health problems	-Reduce the subjective stress level of healthy people -Improvement of the subthreshold level of stress-related anxiety/depression of healthy people	7	1,146	-	0%	−0.14 (−0.27, −0.01)
(24)	Effectiveness of Probiotic, Prebiotic, and Synbiotic Supplementation to Improve Perinatal Mental Health in Mothers: A Systematic Review and Meta-Analysis	54	MEDLINE (Ovid interface), EMBASE (Ovid interface), CINAHL Plus with Full Text (EBSCOhost interface), Cochrane Central Register of Controlled Trials (Wiley interface, which also includes ClinicalTrials.gov and the WHO International Clinical Trials Registry Platform) Scopus, Web of Science Core Collection, and BIOSIS (Web of Science Platform)	Pre/probiotics or synbiotic compared with no treatment/placebo	Grading of Recommendations Assessment, Development and Evaluation (GRADE) framework	Pregnant women with uncomplicated pregnancies	- Reduced anxiety scores in the STAI-6 questionnaire by almost 1 point at the end of follow-up	3	543	-	0%	−0.99 (−1.80, −0.18)
(25)	Probiotics for Alzheimer’s Disease: A Systematic Review	22 (18 animal studies and 4 clinical trials)	PubMed, Semantic Scholar, Nature, and Springer link	Probiotic compared with a control (placebo or standard treatment)	-	Patients with AD	-Improved Mini-Mental State Examination score. - Reduced serum high-sensitivity C-reactive protein (hs-CRP). - Reduced serum triglyceride. - Reduced serum MDA. - Effects on total antioxidant capacity. - Higher TYM score, cognitive function - Increased serum GSH. - Decreased serum 8-OHdG. - Lower concentration of fecal zonulin. - Increased Faecalibacterium - Prausnitzi in fecal. - Higher level of kynurenine in serum. - Higher level of neopterin and nitrite. - Higher RNA level in fecal bacteria	4	192	-	-	-
(26)	Prebiotics and probiotics for autism spectrum disorder: a systematic review and meta-analysis of controlled clinical trials	3	PubMed, Web of Science, Embase, and Cochrane Library	Pre or probiotic compared with placebo	modified Jadad scale	ASD patients	-ADOS-CSS: Total ADOS Calibrated Severity Score. -6-GSI: 6-Gastrointestinal Severity Index. -CBCL: Child Behavior Check List. -ATEC: Autism Treatment Evaluation Checklist. -CGI-I: Clinical Global Impression-Improvement	3	144	Severity of overall ASD symptoms	0%	−0.23 (−0.56,0.11)
										Severity of GIT issues in ASD	85%	−1.14 (−3.56,1.31)
										Comorbid psychopathlology in ASD	0%	−0.06 (−0.37,0. 25)
(27)	A Systematic Review of the Effect of Probiotic Supplementation on Schizophrenia Symptoms	3	PubMed, Medline, Embase, Google Scholar, ClinicalTrials.gov, Clinical Trials Register of the Cochrane Collaboration Depression, Anxiety and Neurosis Group (CCDANTR), and Cochrane Field for Complementary Medicine databases	Probiotic compared with placebo	Cochrane Collaboration’s tool	patients with at least moderately severe psychotic symptoms of SCZ	PANSS (Positive and Negative Syndrome Scale)	3	172	-	0%	−0.09 (−0.38,0.20)

Optimal balance of intestinal microbiota and strengthening of ENS and vagus nerve with the presence of pro-and prebiotics alter and increase metabolites such as tryptophan and SCFAs that directly affect brain function, and the secretion level of some brain factors such as gamma-aminobutyric (GABA), serotonin/5 hydroxy tryptamine, brain-derived neurotrophic factor, and dopamine, ultimately affect mental disorders (2–5). The hypothalamic–pituitary–adrenal tension feedback, which regulates mood and emotion, is weakened by some probiotics, dropping corticosteroid amounts. The immune system, under the influence of pro-and prebiotics, causes the production and secretion of pro-inflammatory cytokines; then, by affecting the nerves and the hormonal system, the amount of inflammation in the target tissue, which is the brain, is reduced (3, 5). Studies have shown that the use of combined probiotics (e.g., *Lactobacillus*, *Enterococcus*, and *Bifidobacterium*) together with prebiotics (e.g., resistant starch, and inulin), called synbiotic products, produces a high level of neurotransmitters and neuropeptides, e.g., GABA and brain-derived neurotrophic factor, improving CNS function, counting psychiatric disease-related functions, such as anxiety, depression, stress, and memory ability (14, 15, 28). Table 2 shows some of the studies that have investigated the relationship between pro and prebiotics and mental disorders.

**Table 2:** The effect of pro and prebiotics on some mental disorders.

Ref	Probiotic strain(s)/Prebiotic	Dose of probiotic/prebiotic	Carrier	Model	Duration	Effect
(29)	*L. acidophilus*, *L. casei*, *L. fermentum*, and *B. bifidum*	2 × 10^9^ CFU/g of each strain	Milk (200 mL/day)	Human	12 weeks	Improving cognitive function in AD patients
(30)	A mixture of lactobacilli (*L. acidophilus*, *paracasei, plantarum*, *bulgaricus*, *delbrueckii*), bifidobacteria (*B. longum*, *breve*, *infantis*), and Streptococci (*S. salivarius*, *thermophilus*)	8 × 10^8^ CFU/g of lactobacilli,9 × 10^10^ of bifidobacteria, and 20 × 10^10^ Streptococci	VSL#3 (VSL Pharmaceuticals Inc., USA)	Human (case report)	4 weeks	Improvement of the core symptoms of ASD in a 12 years old child
(31)	*L. acidophilus*, *L. fermentum*, and *B. lactis*	1 mL of water containing 10^10^ CFU/g of the three bacteria	Drinking water (1 mL/day)	Rat	2 weeks	Improvement of stress-dependent behavioral disorders and the interaction between HPA and gut-brain-microbiota axes
(32)	*L. fermentum* NS9	10^9^ CFU/mL	Drinking water	Rat	41 days	Reduction of anxiety-like behavior as well as reduction of memory retention disorders caused by ampicillin
(33)	*B. breve* CCFM1025	10^10^CFU/day	Freeze-dried	Human	4 weeks	The probiotic group showed a better antidepressant-like effect than the control group (maltodextrin); so this is a promising strain that reduces depression.
(34)	S. *thermophiles, B. breve*, *B. lactis*, *B. infantis*, *L. plantarum*, *L. paracasei*, *L. helceticus*	9 × 10^11^ CFU/day	probiotic supplement (Vivomixx®, Mendes SA, Lugano, Switzerland)	Human	31 days	Probiotic treatment along with changes in gut microbiota, also reduced depressive symptoms
(35)	*L. plantarum* PS128	3 × 10^10^ CFU/capsule	Capsule (Two a day)	Human	30 days	Daily consumption of probiotics may reduce symptoms of depression, improve sleep quality, and reduce fatigue
(36)	Fructooligosaccharide as prebiotic, and probiotics, include *L. casei*, *L. acidophilus*, *L. bulgaricus*, *L. rhamnosus*, *B. breve*, *B. longum*, and *S. thermophilus*	100 mg fructooligosaccharide, and *L. casei* 3 × 10^8^, *L. acidophilus* 2 × 10^8^, *L. bulgaricus* 2 × 10^9^, *L. rhamnosus* 3 × 10^8^, *B. breve* 2 × 10^8^, *B. longum* 1 × 10^9^, and *S. thermophilus* 3 × 10^8^ CFU/g	Capsule (One a day)	Human	6 weeks	Synbiotic is effective as an adjunctive treatment for moderate depression
(37)	Fructan	The amount of fructan received by each person from the number of times consumed and the fructan content of meals	Food consumed by each person	Human (cohort study)	-	A higher dietary intake of fructan is associated with a reduced risk of clinical Alzheimer’s disease in people aged 65 years and older
(38)	Foods containing prebiotics (such as cereals, bread, root crops, milk products, and vegetables)	Amount of consumption of foods containing prebiotics by each person	Food consumed by each person	Human (cross-sectional exploratory study)	-	Lower consumption of prebiotic foods has a negative effect on anxiety, stress and depression
(39)	*Bifidobacterium infantis* and a bovine colostrum product (BCP) as a source of prebiotic oligosaccharides	2 × 10^10^ CFU/day of probiotics and 0.15 g/lb. body weight per day of colostrum powder	milk, juice, yogurt, or ice cream	Human	12 weeks	Particular aberrant behaviors were reduced in some autistic children treated with a combination of probiotics and prebiotics or prebiotics alone

## 4. Portrayal of the effect of pro-and prebiotics on neurological disorders

### 4.1. Impact of pro-and prebiotics on anxiety

Epidemiological investigations have demonstrated that anxiety disorders are the main causes of functional impairment. A number of anxiety disorders include panic disorder, social anxiety disorder, obsessive–compulsive disorder, generalized anxiety disorder, post-traumatic stress disorder, and phobias (40).

Current studies have signified that pre-and probiotic supplementation has a potential impact to improve symptomology in mental ailments (41, 42). Prebiotics reach the colon, and GM can ferment them. On the other hand, prebiotics is the nutrient source for probiotics settling in the colon, and this cooperation surely improves GIT functionality. Specific pro-and prebiotics can confront infections and attenuate the risk of general diseases such as mental disorders (5). The specific lactic acid bacteria such as *Lactobacillus rhamnosus* GG, *Lactobacillus casei*, *Lactobacillus plantarum*, and *Lactobacillus johnsonii*; or *Bifidobacteria* such as *Bifidobacterium bifidum* Bb12, and *Bifidobacterium lactis* or some yeasts like *Saccharomyces cerevisiae* Var. *boulardii* are the main members of probiotics (43).

Clinical researches have detected some psychobiotics with a good antidepressant, and anti-anxiety impacts. These ingredients can regulate GIT microbiota and improve the microbiota–gut–brain axis (20, 21, 44, 45). For example, the latest animal model research indicates that probiotics (such as *Lactobacillus* and *Bifidobacterium* genera) can effectively decrease anxiety-like behaviors in mice or rats assessed in the open field, the increased plus-maze, the light–dark box, and conditioned defensive burying. In addition, probiotics reduce plasma or serum corticosterone levels after severe stress. It is imagined that probiotics have anxiolytic-like impacts through vagal effects on the periaqueductal gray, central nucleus of the amygdala, caudal solitary nucleus, and bed nucleus of the stria terminalis. More investigations are needed to indicate the neurochemical anatomy under GM exerting effects through vagal or nonvagal pathways (44).

The results of an intervention study that was conducted for 4 weeks showed that GOS prebiotic supplement may be effective in improving pre-clinical anxiety indices (46). Moreover, in a meta-analysis it was shown that pro-and prebiotic supplement, as isolated therapies, incurred non-statistically significant results (45). Furthermore, the anxiety-reducing effects of probiotics in populations with anxiety was documented significantly (47). The probiotic impacts on the improvement of anxiety were exerted through several mechanisms, such as promoting the ENS or the immune system’s stimulation through the bacteria, as well as affecting the psychophysiological markers of depression and anxiety in three different ways. They can decrease systemic inflammation and control the hypothalamic–pituitary–adrenal axis stress response. In addition, these substances induce the secretion of molecules such as neurotransmitters, proteins, and SCFAs can have a direct effect on the immune system (48).

### 4.2. Impact of pro-and prebiotics on depression

Regarded to WHO research, major depressive disorder leads to disability worldwide. GM is a factor that can be effective in depression and exerts its effect through the microbiota–gut–brain axis (1). Gut dysbiosis impairs mental health and mental health disorder interrupt gut microbiota. Depressive symptoms are usually associated with GIT disorders such as inflammatory bowel disease, metabolic syndrome, and irritable bowel syndrome. The concurrent occurrence of mental and GIT disorders enhances disease progression and intensifies the occurrence of poorer consequences, whereas, treatment of one of these two conditions can reverse the risk of the other. Moreover, the pathogenesis of depression is comorbid with alterations in the composition of GM (49).

Recent investigations have revealed probiotics positively affect individuals with pre-existing depressive symptoms, while, in healthier populations, mood symptoms are less significantly affected (5, 20, 50). Investigating the behavioral changes caused by LPS-induced in a rodent model to investigate the relationship between the absence of GM and neuroinflammatory mechanisms has shown that the activation of pro-inflammatory mechanisms, the activation of the raphe nucleus, and depression-like behaviors are affected by GM function (51). Overall, the evidence represents that GM plays a potential role in the pathogenesis of depressive behavior and may be an antidepressant agent. In addition, molecules derived from microorganisms, such as SFCAs, indoles, bile acids, neurotransmitters, lactate, choline metabolites, and vitamins could be largely effective in stimulating emotional behavior. The neuroactive molecules (such as dopamine, tryptamine, GABA, acetylcholine, 5-hydroxytryptamine/serotonin 5-HT, L-dopa, norepinephrine and histamine) are directly produced by the microbiome (52). Also, any changes in intestinal flora suppress hippocampal brain-derived neurotrophic factor expression in the neurons in the cortex and hippocampus leading to depression (53).

The intestinal microbiota can affect the brain tissue directly by regulating the secretion of hormones from brain-gut peptide production, intestinal endocrine cells, corticotropin, corticotropin-releasing factor, leptin and adrenocortical ketone. Furthermore, it was released that probiotics can play a role in changing the sensitivity of the intestinal tract, regulating the stimulation threshold of intestinal neurons and the secretory function of intestinal cells, maintaining the ecological stability of GM, and then influencing the CNS and improving depression (54). Prebiotics can also up-regulate the expression of the specific gene in the hippocampus and hypothalamus tissue, promote propionic acid and cecal acetic acid and reduce the isobutyrate value which is associated with behavioral improvement. Nevertheless, the mechanism of the microbiome-gut-brain interaction is still not fully elucidated (55).

On the other hand, some investigations have proved that pre-and probiotics have potential impacts on depression-like behavior through restoring cortisol values, attenuating the inflammatory mediators, and as well as regulating serotonin and CNS transmitters synthesis (56, 57). For instance, Schmidt et al. (57) reported that the awakening response of salivary cortisol declined significantly after B-GOS (Bimuno®-galacto-oligosaccharides) intake compared to placebo. Additionally, in a dot-probe task, it was observed that after taking B-GOS, there is a decrease in attentional vigilance toward negative information versus positive information. No significant results were found in healthy participants to intake FOS for 3 weeks (57). Zagórska et al. (20) revealed that probiotic consumption significantly reduced the symptoms of patients with depression after 8 weeks (20). Similarly, the meta-analysis of 34 controlled clinical trials, statistically showed that probiotics have significant effects on depression; however, the prebiotics did not differ from placebo for depression (49). In addition, treatment with *L. paracasei* strain Shirota for 12-week in eligible patients with bipolar disorder can reduce depression severity significantly evaluated by the Hamilton Depression Rating Scale (58).

### 4.3. Impact of pro-and prebiotics on stress

Stress is a major agent of the occurrence of horrible diseases such as heart disease. The healthy ways are believed to attenuate stress. The pro-and prebiotics potential effects on managing stressful conditions are very intriguing. The literature reviews have represented that GM has roles in the regulation of stress. The microbiome-gut-brain axis is a complex and bidirectional network that exists between the CNS and GM and any imbalance in this axis, induces various kinds of mental health disorders such as stress (20).

The bacteria are removed from the mucosa through the inherent and adaptive immune systems. Bacterial metabolites can induce the discharge of neuropeptides and other gut hormones from enteroendocrine cells. GM considerably affects the progress and sensitivity of the hypothalamic–pituitary–adrenal axis in responding to stressors. The grade of psychological stress may be progressed by dysbiosis of GM. Conversely, chronic psychological stress may exacerbate the degree of dysbiosis. It was demonstrated both probiotics and antibiotics can decrease psychological stress retorts (5, 59). Various prebiotics have enhanced the stress-protective microbial organism’s activity and growth. Therefore, prebiotics in the diet increases the bacterial species to produce lactic acid and butyrate (stress-protective microbial species) and maintain the host from the neurobiological, behavioral, and physiological effects of stress (23, 60).

The long-term administration of a CP2305 tablet (containing heat-inactivated, washed *Lactobacillus gasseri*), in healthy young adults, significantly decreased stress and stress-induced instability in GM through the elevation of *Streptococcus* spp. the decline of *Bifidobacterium* spp. in feces (23). In addition, it was found that *Bifidobacterium longum* 1714™ modulated resting neural activity, enhanced mental vitality, and attenuated mental fatigue which leads to neural response modulation during stress (61). A diet containing prebiotics and regular exercise can be appropriate and practical strategies to enhance stress-protective bacteria and resistance to the neurobiological effects of stress (60). On the other hand, (B)-GOS supplementation decreased the neuroendocrine stress response and improved emotional attention in healthy participants after 3 weeks (57).

### 4.4. Impact of pro-and prebiotics on sleep

Sleep disorders as a type of circadian rhythms sleep–wake disruption are characterized by insomnia or excessive sleepiness. People working night shifts have a circular rhythm disorder that generally shows less sleep time than the evening and day shift workers. The literature review has found probiotic administration can improve sleep quality which is related to balancing GM (62). The significant bidirectional connection between GIT and CNS (the gut-brain axis) plays a major role to regulate the GM composition. Therefore, probiotic supplementation may help to improve sleep quality by maintaining the balancing of the GM environment. Furthermore, probiotics promote the production of free tryptophan in the CNS, and promote melatonin formation from serotonin can regulate circadian rhythm (63). The prebiotics also can efficiently affect mental health and ameliorate cognitive function and sleep/wake cycle disruptions (2).

A clinical trial investigating which was conducted to evaluate the *Lactobacillus casei* strain *Shirota*/LcS effect on sleep quality under psychological stress, reported a significant positive effect of LcS supplementation and sleep quality. The results showed that the daily administration of LcS may maintain the quality of people’s sleep during a period of increasing stress (64). In another investigation, consumption of a tablet containing heat-inactivated washed *Lactobacillus gasseri* CP2305 in healthy adults decreased sleep disorders (65). Similarly, a double-blind, placebo-controlled study with a probiotic mixture (containing *L. plantarum* LP01, *Lactobacillus fermentum* LF16, *L. rhamnosus* LR06, and *Bifidobacterium longum* BL04) for 6 weeks, significantly improved sleep quality in the probiotic group (66). Recently conducted meta-analytic studies indicate that probiotic supplements could be significantly effective in improving perceived sleep quality (21, 22).

### 4.5. Impact of pro-and prebiotics on Alzheimer’s

Alzheimer’s disease (AD), recognized as the most prevalent form of dementia currently affects around 50 million cases worldwide (67). At first, it was defined as a clinic-pathology status. Nowadays it is referred to as Alzheimer’s clinical syndrome with a range of clinical manifestations and a multifactorial etiology that has several pathobiological subtypes. The basis of the definite diagnosis of AD is through pathological examinations and includes; observation of extracellular plaques with depositions of β-amyloid/Aβ, presence of Aβ in the brain vessels (cerebral amyloid angiopathy (CAA)), and protein forming neurofibrillary tangles associated with intraneural accumulation of abnormal hyperphosphorylated tau protein (67, 68). AD is a progressive neurodegenerative disorder characterized by memory loss, and problems with thinking, language, and problem-solving abilities. As it is an age-dependent situation, the problem will grow as the average age of the population increase. Another background factor is sex; AD is more common in women (68). During this review, we will discuss how the pathobiology of the disease can be described by the gut-brain microbiota connections.

Generally, we lack information about the exact cause of AD; however, there are some hypotheses about the etiology of the disease; (i) amyloid theory which has long been the main theory: Accordingly, alterations in the process of the Aβ cycle cause accumulation of Aβ protein in the brain. These plaques are harmful to the neurons and also cause oxidative damage. The generated Aβ cycles, in turn, induce forming neurofibrillary, and phosphorylation of tau protein which leads to further damage to the neural system (69). (ii) Presently, neurodegeneration caused by various mechanisms is considered to describe AD. Problems in the hemostasis of calcium, amyloid accumulation, imbalance of neurotransmitters, neuro-inflammation and astrocyte activation, and brain atrophy are some of the suggested mechanisms (19).

As discussed above, the neuropathology of AD has long been considered only a brain disease; however new evidence is supporting the idea of the effects of other organs in developing AD. Mainly the role of GM in the normal function of the brain and nervous system has been broadly studied. The findings suggest that GM can affect the structure and function of the brain directly. It can also change the immunity and behavior of the host, which indirectly affects brain function. There are some data available from experimental and clinical studies showing altered microbiome in neurodegenerative diseases such as AD. There are some mechanisms proposed for the effects; the transformed microbiome induces the release of neurotransmitters and pro-inflammatory factors leading to the increased permeability of the blood–brain barrier which in turn causes augmented neuro-inflammatory reactions and amyloid production and accumulation in the brain tissue. The dyes-biome allows the entrance of bacterial amyloid, LPS, and some toxic molecules in peripheral blood circulation and lately in the brain which in turn cause abnormal changes in the brain. Neurodegeneration may also be induced by dysfunction of the immune system related to the abnormal microflora. It should be mentioned that it is a chronic situation and the pathologic changes begin 10–20 years before the manifestation of the clinical disease. It can be concluded that restoring GM in patients with AD can no doubt slow down the progression of abnormal changes in the brain by reducing amylogenesis, and inflammation (19).

It is obvious that pro- and prebiotics may successfully be applied to cure patients with AD. For instance, the probiotic beverage containing *L. acidophilus*, *L. fermentum*, *L. casei*, and *B. bifidum* for 12 weeks significantly improved Mini-Mental State Examination score in 60 patients with AD, with a mean age of 80 (5). Gene profiling studies demonstrated that *Bifidobacterium breve* A1 can suppress inflammation in the hippocampus of the brain and also immune-reactive genes induced by amyloid (19). Lactopeptides and tryptophan-related dipeptides in fermented dairy products showed positive effects on memory and cognition function. In addition, there are some evidence which show consumption of dairy products such as cheese and milk reduce the risk of dementia and cognitive dysfunction (19). One systematic review conducted on the effects of the probiotics on AD reached plenty of evidence about promising effects of probiotics in improving the progression of the disease including *in vivo* studies and clinical trials. No side effects were reported (25).

### 4.6. Impact of pro-and prebiotics on autism spectrum disorder

ASD is a condition characterized by difficulties in social communication and interaction, repetitive and limited patterns of interests and behaviors, and changes in sensory processing related to neurobehavioral and neurodevelopment abnormalities (70). The results of ongoing research show that it is a growing concern with an increasing prevalence all over the world. Prevalence estimates published 10 years ago suggest around 100/10,000 morbidity with the male sex about 4 times more likely to get the disease (71). Due to the complex nature of the disease, it is known to have several etiological backgrounds including; anatomical changes in the brain, genetic abnormalities, and neurochemical dysfunctions. The altered pathways of many neurotransmitters including serotonin, dopamine, N-acetyl aspartate, oxytocin, GABA and glutamate, acetylcholine, arginine-vasopressin, vitamin D, melatonin, orexin, and opioids are supposed to have a role in the disease mechanism. However, the complex relationship between the abnormal neurotransmitters and the specific interaction system underlying the disease has not been recognized yet (70).

There are some evidences about the potential effects of GM on the pathogenicity of Autism. There is a high comorbidity of GIT symptoms such as abdominal pain, diarrhea, constipation, and the disease, and this, in turn, increases the behavioral problems in patients. The gut-brain interactions are related to the pathophysiology of ASD via the population and function of GM. It has been demonstrated that gut bacterial profile is different in patients with ASD compared to the normal controls. However, the altered microbiota may be the result of the special lifestyle of the patients such as diet and bowel habits. Based on the findings, the idea of the therapeutic effects of changing GM on ASD was developed. During a study, GM from patients with ASD was transferred to germ-free mice which induce autism symptoms such as repetitive behavior and decreased communication and locomotion. In addition, treatment with bacterial metabolites like 5-aminovaleric acid which is depleted in ASD patients can improve the function of the prefrontal cortex (related to social cognition) and consequently repetitive and social behavior. Among various therapeutic candidates to modulate the gut-brain axis in ASD, pro-and prebiotics have drawn special attention (28).

Several studies have been conducted to assess the effects of pro-and prebiotics on ASD. The main endpoints were ASD-related symptoms and GI wellbeing. Various strains of *Lactobacillus* such as *L. acidophilus*, *L. plantarum*, *L. paracasei*, as well as *Bifidobacterium* had been administered. Hydrolyzed guar gum, FOS, and maltodextrin were also applied to the patients as prebiotics. Some RCTs found no significant difference between probiotic and placebo groups regarding behavioral problems and symptoms severity after completion of the intervention. Other studies with significant differences between placebo and control groups were subject to bias distorting the effect. It can be concluded that the effect of probiotics on ASD symptoms has not been proven yet. However, studies on the effects of prebiotics and synbiotics show the beneficial effect of the treatment to improve some scales of the ASD-related symptoms. For instance, GOS containing prebiotic supplement (Bimuno®) can reduce anti-social behavior (72), and the combination of prebiotic oligosaccharides and *B. longum* subsp. *infantis* UCD272 on the lethargy of the patients showed positive effect (39).

According to the results of a systematic review on the RCTs, four of the trials showed no changes after consumption of probiotics. A significant reduction in GIT symptoms was demonstrated in two of the trials, and it was known to be associated with ASD behavioral symptoms. The main finding of the studies was the improvement in GIT symptoms such as constipation, diarrhea, and stool smell in the prebiotic compared to the control group. It should be noted that the treatment duration in the studies on prebiotics and synbiotics is longer than the probiotic studies, and it may be the reason for the observed effects in the prebiotic studies. Also, these studies are accompanied by various outcomes and comparisons such as sub-group analysis which increases the chance of statistically significant difference. So, some of the significant results may be simply due to the chance and not the real effects of the administered compounds. It can be concluded that we cannot still say for sure that probiotics, prebiotics, or synbiotics can make a positive change in ASD patients (28).

### 4.7. Impact of pro-and prebiotics on schizophrenia

Schizophrenia (SCZ) is a kind of psychiatric disorder with a global age-standardized prevalence of 0.28% and no sex difference in prevalence. The prevalence does not vary extensively across the countries. Though the low prevalence of the disease, it has a substantial burden on society due to the poor recovery outcome, and the decreased life expectancy and life quality. Suicide attempts and comorbid diseases (coronary heart disease, type II diabetes, respiratory, and malignancies) are from the problems of the individuals with SCZ (73). The symptoms are categorized into three main groups; positive, negative, and cognitive. Positive symptoms or the presence of psychotic symptoms are more responsive to the antipsychotic medication treatment than negative (e.g., social withdrawal) and cognitive (e.g., diminished abstract thinking) symptoms. The etiology of the disease has not been fully understood yet, however genetic and environmental factors are supposed to interact to induce the symptoms. Availability of proper medications is very important as early treatment, monitoring, suitable psychological management, and social support may lessen the symptoms or even lead to partial or full remission (74).

As discussed previously, there is much evidence on the effects of GM on brain functions and subsequently behavior and psychiatric problems. The mechanisms may also involve in SCZ. Studies on animal models suggest that some SCZ-associated behaviors such as social behaviors, cognition, and mood alterations can be influenced by GM. However, clinical studies on humans are still limited (74). The studies are focused on two main backgrounds; comparing the microbiome of the patients with SCZ and the healthy controls, and clinical trials to detect any therapeutic advances in the administration of pro-and prebiotics for schizophrenia. It has been demonstrated by several studies that the level of the family Lachnospiraceae is lower in individuals with SCZ compared to the healthy population which involves protecting the integrity of the intestinal barrier and producing beneficial compounds. However, the results of this type of studies are subject to biases due to the effects of psychiatric treatments and lifestyle on the microbiome (74).

Albeit promising effects of the pre and probiotics in experimental designs, a systematic review of the trials till 2018 revealed no beneficial effects of probiotics on SCZ on meta-analysis. The authors concluded that regardless of the positive effects of the probiotics on bowel movement and ameliorating the metabolic effects of antipsychotic medications the administration of probiotics for SCZ is not recommended (27). We found no systematic review of the effects of prebiotics but the results of the trials imply potential beneficial effects. In one study, application of oligofructose-enriched inulin (OEI) increased serum butyrate in SCZ patients (75). Another prebiotic, lactosucrose, altered the fecal flora followed by improvement in the intestinal and psychotic symptoms of the patients (76).

## 5. Conclusion and future perspectives

The communication between GIT and the brain has long been well known. The direct neural signals and indirect hormonal and enzymatic connections are supposed to be responsible for the mutual effects. The idea developed to the application of pre-, pro-and synbiotics to modulate the CNS during mental disorders as a novel and natural treatment with very limited potential side effects. In this review we presented promising findings on the effects of pre-, pro-and synbiotics on a variety of mental disorders especially anxiety, depression, stress, sleep, and AD. Despite some studies on the positive effects of pre-, pro-and synbiotics on the other mental conditions including SCZ and ASD, the available data is not enough to support the idea of the application of such therapies for the above disorders. It is obvious that we need to expand our knowledge on this subject by conducting well design clinical trials using various kinds of pre-, pro-and synbiotics in well-defined and -as far as possible- large populations to get more specific and more reliable results. The present evidence is attractive enough to go ahead and design special formula of pre-, pro-and synbiotics for different mental disorders. This may also be accompanied by testing different drug regimens containing standard treatments and pre-, pro-, or synbiotics. In conclusion, it can be said that it is time to introduce a new generation of specific drugs based on the pre-, pro- and synbiotics for a variety of mental disorders. A need that should be met through conducting appropriate and rigorous research plans.

## Author contributions

FA and HP conceived the idea. FA, MN, HP, SJ, SAS, and EM wrote sections of the manuscript. All authors contributed to the manuscript revision, read, and approved the submitted version.

## Conflict of interest

The authors declare that the research was conducted in the absence of any commercial or financial relationships that could be construed as a potential conflict of interest.

## Publisher’s note

All claims expressed in this article are solely those of the authors and do not necessarily represent those of their affiliated organizations, or those of the publisher, the editors and the reviewers. Any product that may be evaluated in this article, or claim that may be made by its manufacturer, is not guaranteed or endorsed by the publisher.

## Abbreviations

GIT: gastrointestinal tract
CNS: central nervous system
GM: gut microbiome
GOS: galactooligosaccharides
FOS: fructooligosaccharides
ENS: enteric nervous system
SCFAs: short-chain fatty acids
LPS: lipopolysaccharides
GPCRs: G-protein coupled receptors
TLRs: Toll-like receptors
GABA: gamma-aminobutyric
PSQI: Pittsburgh Sleep Quality Index
AD: Alzheimer’s disease
ASD: Autism spectrum disorder
SCZ: Schizophrenia
